# Heterogeneity-aware personalised federated learning for household energy forecasting on multi-source real-world smart meter data

**DOI:** 10.1038/s41598-026-53020-6

**Published:** 2026-06-01

**Authors:** Pushpak Das, Stuti Pandey, Chandrasen Pandey, Deepak Kumar

**Affiliations:** 1 National Institute of Technology Meghalaya, Saitsohpen, Sohra, East Khasi Hills District, Meghalaya 793108 India; 2 Department of Artificial Intelligence and Machine Learning, Manipal University Jaipur, Jaipur, Rajasthan India; 3 School of Computer Science, University of Petroleum and Energy Studies (UPES), Dehradun, Uttarakhand India

**Keywords:** Energy forecasting, Federated learning, Personalised federated learning, Non-IID data, Smart meters, Household load prediction, Edge intelligence, Time-series forecasting, Energy science and technology, Engineering, Mathematics and computing

## Abstract

Residential and building-level electricity forecasting is increasingly based on smart-meter streams, but direct data pooling is often difficult because load traces are private, geographically dispersed and statistically different across households and buildings. This paper studies the effect of such client heterogeneity in federated energy forecasting. A multi-source benchmark is constructed by combining processed Smart, CU-BEMS, UCI Household and AMPds clients, giving 22 usable clients and 1,522,510 observations at 15-minute resolution. The benchmark compares non-federated baselines (Persistence, Ridge, LocalOnly and Centralised training), standard federated baselines (FedAvg, FedProx and FedPer) and the proposed HAPFL framework over 1-step, 12-step and 24-step forecasting horizons. HAPFL separates a shared temporal encoder from client-specific prediction heads and combines proximal stabilisation, latent prototype alignment and difficulty-aware aggregation. In the reported benchmark protocol, HAPFL gives the lowest mean MAE and the highest mean $R^2$ at all three horizons. At horizon 1, mean MAE improves from 0.208 to 0.196 and mean $R^2$ improves from 0.741 to 0.769 relative to centralised training, while the worst-10% client MAE is reduced from 0.301 to 0.274. At horizons 12 and 24, the corresponding MAE reductions over centralised training are 6.11% and 7.60%, respectively. The results indicate that vanilla federated averaging is not adequate for strongly non-identically distributed energy clients, while personalised and heterogeneity-aware federated learning is a more suitable direction for decentralised household and building load forecasting.

## Introduction

Fine-grained electricity forecasting at household and building level is important for demand response, tariff planning, storage scheduling and reliable operation of modern distribution systems^1^. The wider use of smart meters has made such forecasting technically feasible and learning-based models have reported strong predictive performance in centralised settings^2,3^. At the same time, smart-meter traces may reveal occupancy patterns, appliance use and daily behaviour. For this reason, it is not always acceptable to pool raw consumption data across homes, buildings, utilities, or institutions^4^. The practical question is therefore not only how to forecast accurately, but also how to learn from distributed data without moving raw records to a central store.

Federated learning (FL) addresses part of this issue by allowing clients to train a model collaboratively while keeping raw data local^5,6^. In the energy domain, this is attractive because electricity traces are often held by many physically separated clients. However, data locality alone does not make the learning problem easy. Smart-meter clients are usually non-identically distributed (non-IID): they differ in load magnitude, occupancy routine, appliance mix, weather exposure, source type, sample length and data quality. In such settings, a single globally averaged model can be dominated by larger or easier clients, leaving difficult clients under-served^7,8^.

This issue is particularly important for household and building energy forecasting because heterogeneity is part of the problem rather than a small disturbance. Residential clients and institutional buildings have different demand rhythms and even similar households can have very different short-term patterns. Recent work has therefore started moving beyond simple model averaging by considering asynchronous training, adaptive aggregation, robust aggregation and personalised model components^9–14^. These studies show that FL is feasible for energy applications, but they also show that the central challenge is the handling of heterogeneous clients.

Motivated by this setting, this paper studies household and building energy forecasting under a realistic multi-source client pool. We construct a federated forecasting testbed from processed Smart, CU-BEMS, UCI Household and AMPds data and evaluate classical, local, pooled centralised and federated baselines across short and medium forecasting horizons. We then propose HAPFL, a heterogeneity-aware personalised federated forecasting framework. The method separates shared temporal representation learning from client-specific prediction, so that common sequential structure can be learned collaboratively while local load characteristics are retained in personalised heads. To stabilise training under non-IID data, the local objective includes proximal regularisation and latent prototype alignment. At the server, aggregation is adjusted using both client data volume and validation difficulty.

The main contributions of the paper are as follows.

1. A multi-so is formed with 22 usable clients and 1,522,510 observations at 15-minute resolution, covering both household and building settings.
2. The empirical comparison shows that vanilla FedAvg is weak under this level of heterogeneity, whereas stabilised and personalised FL methods are more competitive.
3. HAPFL is introduced as a personalised FL framework with a shared temporal encoder, client-specific prediction heads, proximal stabilisation, latent prototype alignment and difficulty-aware aggregation.
4. The reported benchmark results show improvements in mean MAE, mean $R^2$ and difficult-client performance over Persistence, Ridge, LocalOnly, Centralised training, FedAvg, FedProx and FedPer.
5. The study reports repeated-seed stability, paired client-level testing, source-wise checking and difficult-client evaluation to support the benchmark results.

The remainder of the paper is organised as follows. Section Related work and research gap reviews the related literature and clarifies the research gap. Section Problem formulation defines the forecasting problem and notation. Section Proposed method: HAPFL presents the proposed HAPFL framework. Section Benchmark construction and experimental protocol describes the benchmark construction and evaluation protocol. Section Results reports the empirical results. Section Discussion discusses implications, limitations and validation requirements, and Section Conclusion concludes the paper.

## Related work and research gap

Early FL work established iterative model averaging as a practical alternative to central data pooling, but the basic formulation assumes that one shared model can represent all participating clients^5^. For energy forecasting this assumption is fragile because smart-meter clients are heterogeneous in both statistical distribution and data volume. FL offers a scalable alternative to heavier privacy-preserving computation such as homomorphic encryption^15^, but privacy of raw data should not be confused with robustness to non-IID learning.

Fekri et al. studied distributed load forecasting with recurrent neural networks on smart-meter data and showed that FL can be competitive with local and pooled alternatives under some settings^9^. Their later FedNorm work introduced asynchronous and adaptive aggregation for smart-meter forecasting, addressing stale updates and non-uniform client participation^10^. Sarmento et al. evaluated a federated CNN–LSTM pipeline on energy forecasting tasks involving Smart, Building Data Genome 2, augmented data and edge-device settings^11^. These works established feasibility, but the question of how to personalise forecasting under strong client heterogeneity remains open.

There is also relevant work outside standard FL. Load profile restoration and robust forecast enhancement methods show that imperfect or incomplete energy predictors can be improved through model correction, side information, or expert aggregation. Li et al. proposed load profile inpainting for missing load data restoration and baseline estimation, demonstrating the role of context-aware reconstruction in energy data quality problems^16^. Incremona et al. studied aggregation of nonlinearly enhanced experts for electricity load forecasting, which is related to the present paper’s motivation that different predictors or client behaviours should not be averaged blindly^17^. These methods are not federated baselines, but they support the broader argument that robustness in energy forecasting often requires correction, weighting, or adaptation rather than a single unqualified predictor.

In general personalised FL, FedProx adds a proximal term to reduce client drift under heterogeneous data and systems conditions^8^. pFedMe uses Moreau envelopes to learn personalised models close to a global reference^18^, SCAFFOLD uses control variates to correct client drift^19^ and Ditto combines global training with personalised local models for fairness and robustness^20^. FedPer keeps shared feature-extraction layers but retains personalisation layers locally^21^. FedNova addresses objective inconsistency caused by uneven local work^22^, while Byzantine-resilient methods such as CBRFL study robustness to unreliable updates in broader FL systems^14^. Energy-efficient data collection and edge association work also underlines that practical FL is constrained by sensing, communication and energy resources^23,24^.

Recent energy-specific personalised FL studies are closer to the present work. Wang et al. proposed personalised federated learning for building energy forecasting^12^. Zhu et al. proposed PF-HoLo for household load prediction with imbalanced historical data^13^. Review articles in renewable-energy FL identify heterogeneity, fairness, communication cost, privacy guarantees and reproducibility as open issues^25^. The present work is positioned in this gap: it evaluates a multi-source household-and-building benchmark and studies a personalised framework that combines local heads, stabilised representation learning, prototype alignment and difficulty-aware aggregation (Table 1).

**Table 1:** Representative literature and the gap addressed in this paper.

Study	Setting	Method family	Personalisation	Energy domain	Main limitation addressed here
Fekri et al.^9^	Smart-meter forecasting	FL with RNN/LSTM	No	Residential load	Demonstrated feasibility, but client heterogeneity is not the main design target.
Fekri et al.^10^	Distributed smart-meter forecasting	Asynchronous adaptive FL	Partial	Residential load	Strong systems contribution; personalised heads are not central.
Sarmento et al.^11^	FL on multiple energy datasets	CNN–LSTM FL	No	Building and energy load	Broad evaluation, but limited explicit personalisation and difficult-client analysis.
Incremona et al.^17^	Forecast aggregation	Nonlinear expert aggregation	Not FL	Electricity load	Shows value of adaptive forecast combination, but not decentralised client learning.
Wang et al.^12^	Building forecasting	Personalised FL	Yes	Building energy	Focuses on buildings; multi-source household-building heterogeneity is not the main focus.
Zhu et al.^13^	Household load prediction	Personalised FL	Yes	Residential load	Handles imbalance, but source-stratified multi-horizon tail behaviour is less explicit.
This work	Multi-source benchmark with 22 clients	Personalised heterogeneity-aware FL	Yes	Household and building load	Jointly reports mean accuracy, horizon-wise degradation and cautious tail-client robustness under non-IID energy forecasting.

## Problem formulation

Consider *K* clients, where each client corresponds to one household or building-floor load stream. Client *k* holds a local multivariate time series

1 $$\begin{aligned} \mathcal {D}_k = \{(\boldsymbol{x}_{k,t}, y_{k,t})\}_{t=1}^{T_k}, \end{aligned}$$

where $y_{k,t} \in \mathbb {R}$ denotes the target power reading at time index *t* and $\boldsymbol{x}_{k,t}$ contains the target history together with engineered calendar covariates. In the present benchmark, all series are aligned to a 15-minute cadence.

Using a sliding window of length *L*, the local forecasting task for client *k* at horizon *h* is to map

2 $$\begin{aligned} \textbf{X}_{k,t}^{(L)} = [\boldsymbol{x}_{k,t-L+1}, \ldots , \boldsymbol{x}_{k,t}] \end{aligned}$$

to the future target

3 $$\begin{aligned} \hat{y}_{k,t+h} = \mathcal {M}_k\!\left( \textbf{X}_{k,t}^{(L)}\right) , \qquad h \in \{1,12,24\}. \end{aligned}$$

The three horizons correspond to 15 minutes, 3 hours and 6 hours ahead, respectively.

In standard FL, the objective is to learn a single set of global parameters $\boldsymbol{w}$ by minimising the weighted empirical risk

4 $$\begin{aligned} \min _{\boldsymbol{w}} F(\boldsymbol{w}) = \sum _{k=1}^{K} \frac{n_k}{\sum _{j=1}^{K} n_j} F_k(\boldsymbol{w}), \end{aligned}$$

where $n_k$ is the number of local training windows at client *k* and $F_k(\boldsymbol{w})$ is the local objective. Equation (4) is standard, but it implicitly assumes that one global parameter vector can serve all clients. The benchmark in this paper tests this assumption directly under mixed household and building clients.

## Proposed method: HAPFL

**Fig. 1:**
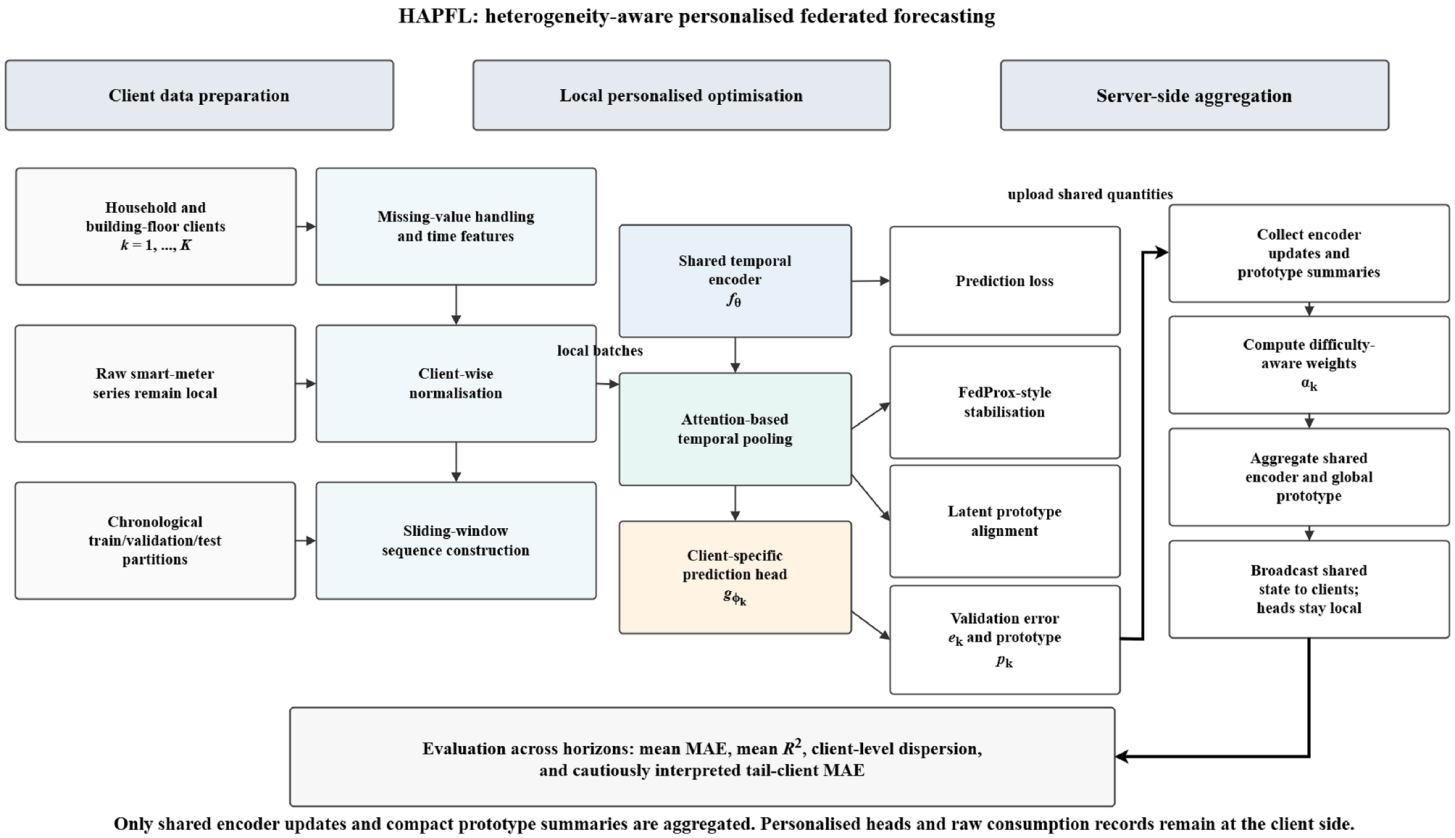
Proposed HAPFL framework for household and building energy forecasting. Each client performs local preprocessing and sequence construction. Local training uses a shared temporal encoder, attention-based pooling and a client-specific head. Only shared encoder updates and compact prototype summaries are sent to the server; raw consumption records and personalised heads remain local.

As illustrated in Fig. 1, the proposed HAPFL framework consists of client-side preprocessing, personalised local optimisation and difficulty-aware server aggregation. The design follows a simple idea: the temporal encoder should learn common sequential structure across clients, while the final predictor should remain flexible enough to reflect each client’s local load pattern.

### Model architecture

The proposed model decomposes the predictor into a shared temporal encoder and a client-specific prediction head:

5 $$\begin{aligned} \hat{y}_{k,t+h} = g_{\phi _k}\big (f_{\theta }(\textbf{X}_{k,t}^{(L)})\big ), \end{aligned}$$

where $f_{\theta }(\cdot )$ is shared across all clients and $g_{\phi _k}(\cdot )$ is personalised. The encoder captures transferable temporal representation, while the head adjusts the forecast to client-specific load level, routine and local seasonality.

In the reported implementation, $f_{\theta }$ is a compact sequence encoder with recurrent temporal processing followed by attention-based pooling. This backbone can be replaced by another sequence model, such as a Transformer-style architecture^26^, without changing the federated optimisation. The important design choice is the parameter split: shared representation learning is collaborative, while the final prediction layer is retained locally. This is consistent with layer-wise aggregation and personalisation ideas in FL^21,27^.

### Local objective with proximal stabilisation and prototype alignment

For a mini-batch $\mathcal {B}_k$ from client *k* during round *r*, the prediction loss is

6 $$\begin{aligned} \mathcal {L}_{k}^{\text {pred}}(\theta ,\phi _k) = \frac{1}{|\mathcal {B}_k|}\sum _{(\textbf{X},y)\in \mathcal {B}_k} \big (y - g_{\phi _k}(f_{\theta }(\textbf{X}))\big )^2. \end{aligned}$$

To reduce client drift, a FedProx-style penalty is added on the shared encoder relative to the current server model $\theta ^{(r)}$:

7 $$\begin{aligned} \mathcal {L}_{k}^{\text {prox}}(\theta ) = \frac{\mu }{2}\left\Vert \theta - \theta ^{(r)} \right\Vert _2^2, \end{aligned}$$

where $\mu \ge 0$ controls stabilisation strength.

Let

8 $$\begin{aligned} \textbf{p}_k^{(r)} = \frac{1}{|\mathcal {B}_k|} \sum _{\textbf{X}\in \mathcal {B}_k} f_{\theta }(\textbf{X}) \end{aligned}$$

denote the local latent prototype and let $\bar{\textbf{p}}^{(r)}$ denote the running global prototype received from the server. Prototype alignment is imposed through

9 $$\begin{aligned} \mathcal {L}_{k}^{\text {proto}}(\theta ) = \lambda \left\Vert \textbf{p}_k^{(r)} - \bar{\textbf{p}}^{(r)} \right\Vert _2^2, \end{aligned}$$

where $\lambda \ge 0$ controls alignment strength. This term does not force all clients to have identical latent representations. It discourages unstable local drift while leaving downstream personalisation to the client heads.

The full local objective is therefore

10 $$\begin{aligned} \mathcal {J}_k(\theta ,\phi _k) = \mathcal {L}_{k}^{\text {pred}} + \mathcal {L}_{k}^{\text {prox}} + \mathcal {L}_{k}^{\text {proto}}. \end{aligned}$$

The shared encoder parameters are uploaded to the server after local training, whereas the personalised heads remain local.

### Difficulty-aware server aggregation

Plain data-size weighting can under-represent difficult clients, particularly when a few clients have large training sets but simpler load patterns. Fairness-aware FL work has shown that client-level resource allocation and client utility should be considered when aggregation is performed^28^. To reflect this in energy forecasting, the server computes a validation error score $e_k^{(r)}$ for each participating client after local training and normalises it within the active set $\mathcal {S}^{(r)}$:

11 $$\begin{aligned} \tilde{e}_k^{(r)} = \frac{e_k^{(r)}}{\frac{1}{|\mathcal {S}^{(r)}|}\sum _{j\in \mathcal {S}^{(r)}} e_j^{(r)}}. \end{aligned}$$

The aggregation weight is then

12 $$\begin{aligned} \alpha _k^{(r)} = \frac{n_k\big (1 + \gamma \tilde{e}_k^{(r)}\big )}{\sum _{j\in \mathcal {S}^{(r)}} n_j\big (1 + \gamma \tilde{e}_j^{(r)}\big )}, \end{aligned}$$

where $\gamma \ge 0$ controls the emphasis on difficult clients. The shared encoder is updated by

13 $$\begin{aligned} \theta ^{(r+1)} = \sum _{k\in \mathcal {S}^{(r)}} \alpha _k^{(r)}\theta _k^{(r+1)}, \end{aligned}$$

and the global prototype is updated as

14 $$\begin{aligned} \bar{\textbf{p}}^{(r+1)} = \sum _{k\in \mathcal {S}^{(r)}} \alpha _k^{(r)}\textbf{p}_k^{(r+1)}. \end{aligned}$$

Only the shared encoder and global prototype are aggregated. The client heads $\{\phi _k\}$ are retained locally and reused in subsequent rounds.

### Relationship to FedAvg, FedProx and FedPer

The proposed framework is linked to common FL baselines as follows.

- Setting *μ =0*, *λ =0* and using full-model aggregation with size-only weights recovers the spirit of FedAvg^5^.
- Setting *λ =0* while retaining the proximal term and full-model aggregation gives a FedProx-style update^8^.
- Retaining shared layers and client-specific heads, but removing prototype alignment and difficulty-aware weighting, yields a FedPer-like strategy^21^.

This nesting helps interpret the empirical comparison, although a complete component-wise ablation requires additional experiments beyond the available benchmark tables.

**Algorithm 1:**
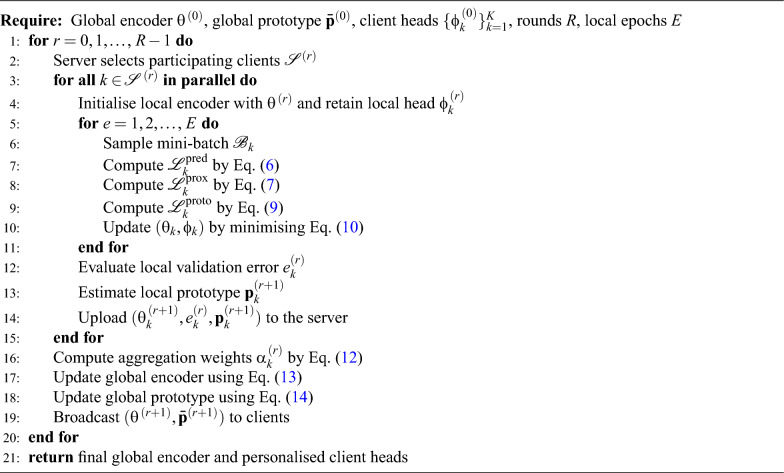
HAPFL Training Procedure

## Benchmark construction and experimental protocol

### Data sources and client definition

The benchmark is formed from four processed data sources: Smart, CU-BEMS, UCI Household and AMPds. Smart provides residential energy traces for sustainable-home research^29^. CU-BEMS provides detailed electricity consumption and indoor-environmental sensor data from a seven-storey office building in Bangkok, Thailand, with measurements for air-conditioning, lighting and plug loads across building zones^30^. AMPds2 provides electricity, water and natural-gas consumption data for a Canadian house at minute resolution over two years^31^. The UCI Household dataset provides individual household electricity consumption data through the UCI Machine Learning Repository^32^.

Each source contributes one or more independent clients. Smart provides seven residential clients. CU-BEMS contributes 13 building-floor clients after excluding one empty file. AMPds contributes one household client and UCI Household contributes one household client. The final benchmark therefore contains 22 usable clients and 1,522,510 observations at 15-minute granularity. Table 2 gives the source-wise breakdown. The benchmark intentionally mixes households and building-floor clients, because this is a direct stress test for non-IID federated forecasting. At the same time, this mixed-source design also means that source-stratified analysis is important for a complete robustness assessment. Treating the CU-BEMS building-floor streams as separate clients is motivated by the federated setting: each metered sub-unit has its own chronology and load profile and can plausibly act as an independently managed participant without raw-data pooling inside the building.

**Table 2:** Benchmark summary after preprocessing and client filtering.

Source	Clients	Observations
Smart	7	551,143
CU-BEMS	13	344,502
AMPds	1	488,513
UCI Household	1	138,352
Total	22	1,522,510

### Preprocessing and input features

All time stamps are converted to a common 15-minute cadence. Missing values are forward-filled when required. Client-wise normalisation is fitted only on the training partition so that validation and test statistics do not leak into training. The input features consist of the target power series and calendar-derived covariates. Exogenous variables such as temperature are not used in the reported benchmark. This keeps the feature layout consistent across all sources, but it also limits comparability with studies that include weather or occupancy measurements.

A rolling window is applied to create supervised sequences. The train/validation/test split is chronological for every client and random shuffling across time is not used. For a leakage-safe implementation, windows that cross split boundaries should be excluded so that no input sequence in validation or test contains target observations from a later evaluation period. The same chronological split policy is intended for every method in the comparison.

### Forecasting setup and baselines

Three forecasting horizons are studied:

- *h=1*: one-step-ahead forecasting (15 minutes),
- *h=12*: 12-step-ahead forecasting (3 hours),
- *h=24*: 24-step-ahead forecasting (6 hours).

The baselines are organised into four groups.

1. **Classical baselines:** Persistence and Ridge on lag windows.
2. **Local learning:** LocalOnly, where each client trains an independent model without collaboration.
3. **Pooled learning:** Centralised, where one model is trained on the pooled training data.
4. **Federated baselines:** FedAvg, FedProx and FedPer.

Additional personalised FL baselines such as pFedMe, SCAFFOLD, Ditto and FedNova are relevant comparators and are discussed in Section Related work and research gap. They are not reported as empirical baselines in the present tables because reproducible benchmark outputs for those methods were not available in the present benchmark record.

This design allows the study to separate three issues: whether collaboration is useful, whether centralised pooling remains a strong competitor and whether personalised FL is more robust than naïve FL.

### Implementation and reproducibility summary

Table 3 summarises the implementation information that is recoverable from the present benchmark record. This table is added to make the protocol easier to reproduce. Hardware, wall-clock training time and communication cost are not included because complete logs for these items were not recorded for the present benchmark.

**Table 3:** Implementation and reproducibility summary for the reported benchmark.

Item	Reported setting
Client pool	22 usable clients from Smart, CU-BEMS, AMPds and UCI Household.
Sampling cadence	Common 15-minute resolution after preprocessing.
Forecast horizons	*h=1*, *h=12* and *h=24*, corresponding to 15 minutes, 3 hours and 6 hours.
Input features	Target load history with calendar-derived covariates; temperature and other exogenous variables are not used.
Normalisation	Client-wise normalisation fitted only on the training partition.
Split policy	Chronological train/validation/test splitting with no random time shuffling.
Model split	Shared temporal encoder with attention-based pooling and local client-specific prediction heads.
Federated update	Shared encoder and prototype summaries are uploaded; raw records and personalised heads remain local.
Validation additions	Five-seed summary, paired client-level testing, difficult-client stress metric and source-group checking.
Open reproducibility items	Full per-client logs, hardware, wall-clock time, communication cost and public processed splits should be released in future work.

### Evaluation metrics

We report Mean Squared Error (MSE), Root Mean Squared Error (RMSE), Mean Absolute Error (MAE), Mean Absolute Percentage Error (MAPE), Symmetric Mean Absolute Percentage Error (sMAPE) and coefficient of determination ($R^2$). For a client with *N* test samples, these are defined as

15 $$\begin{aligned} \text {MSE}&= \frac{1}{N}\sum _{i=1}^{N}(y_i - \hat{y}_i)^2, \end{aligned}$$

16 $$\begin{aligned} \text {RMSE}&= \sqrt{\text {MSE}}, \end{aligned}$$

17 $$\begin{aligned} \text {MAE}&= \frac{1}{N}\sum _{i=1}^{N}\left\lvert y_i - \hat{y}_i \right\rvert , \end{aligned}$$

18 $$\begin{aligned} \text {MAPE}&= \frac{100}{N}\sum _{i=1}^{N}\left\lvert \frac{y_i-\hat{y}_i}{y_i+\epsilon }\right\rvert , \end{aligned}$$

19 $$\begin{aligned} \text {sMAPE}&= \frac{100}{N}\sum _{i=1}^{N}\frac{2\left\lvert y_i-\hat{y}_i \right\rvert }{\left\lvert y_i \right\rvert +\left\lvert \hat{y}_i \right\rvert +\epsilon }, \end{aligned}$$

20 $$\begin{aligned} R^2&= 1 - \frac{\sum _{i=1}^{N}(y_i-\hat{y}_i)^2}{\sum _{i=1}^{N}(y_i-\bar{y})^2}, \end{aligned}$$

where *ε* is a small constant added for numerical stability.

Beyond mean accuracy, we also report

- **Std(MAE):** dispersion of client-level MAE and
- **Worst-10% MAE:** mean MAE over the worst-performing 10% of clients.

With 22 clients, the worst-10% metric corresponds to a very small subset. It is therefore interpreted as a stress indicator for difficult clients rather than as a complete fairness measure.

## Results

All numerical results in this section follow the same benchmark protocol. Along with the main benchmark tables, we also report five-seed mean ± standard deviation and paired client-level testing so that result stability can be checked directly.

### Main benchmark at horizon 1

Table 4 reports the horizon-1 benchmark. HAPFL achieves the best value on every reported metric. Relative to strong centralised training, the mean MAE falls from 0.208 to 0.196 and mean $R^2$ rises from 0.741 to 0.769. Relative to FedAvg, the gain is larger: mean MAE improves from 0.258 to 0.196 and mean $R^2$ improves from 0.582 to 0.769.

The difficult-client result is also relevant. The worst-10% client MAE drops from 0.301 under Centralised and 0.381 under FedAvg to 0.274 under HAPFL. This indicates that the proposed method improves not only the average case but also the difficult tail in the reported benchmark run.

**Table 4:** Benchmark results for horizon *h=1*. Lower is better for MSE, RMSE, MAE, MAPE, sMAPE, Std(MAE) and Worst-10% MAE. Higher is better for $R^2$.

Method	Mean MSE	Mean RMSE	Mean MAE	Mean MAPE	Mean sMAPE	Mean $R^2$	Std(MAE)	Worst-10% MAE
Persistence	0.169744	0.412	0.225	44.0	28.8	0.710	0.091	0.318
Ridge	0.176400	0.420	0.233	47.5	30.2	0.692	0.096	0.333
LocalOnly	0.164025	0.405	0.221	46.1	29.4	0.703	0.089	0.327
Centralised	0.152881	0.391	0.208	40.8	27.0	0.741	0.084	0.301
FedAvg	0.203401	0.451	0.258	61.0	34.6	0.582	0.112	0.381
FedProx	0.147456	0.384	0.202	39.2	26.1	0.751	0.081	0.289
FedPer	0.157609	0.397	0.214	42.5	27.9	0.728	0.086	0.309
HAPFL	0.141376	0.376	0.196	37.1	25.1	0.769	0.075	0.274

### Multi-horizon performance

Tables 5 and 6 show that all methods degrade as the horizon increases, which is expected in realistic time-series forecasting. However, the ranking remains stable in the reported benchmark: HAPFL is best at *h=12* and *h=24* as well. At *h=12*, HAPFL achieves mean MAE 0.246 and mean $R^2$ 0.682, compared with 0.262 and 0.648 for Centralised, 0.254 and 0.664 for FedProx and 0.323 and 0.495 for FedAvg. At *h=24*, HAPFL records mean MAE 0.304 and mean $R^2$ 0.591.

**Table 5:** Benchmark results for horizon *h=12*.

Method	Mean MSE	Mean RMSE	Mean MAE	Mean MAPE	Mean sMAPE	Mean $R^2$	Std(MAE)	Worst-10% MAE
Persistence	0.246016	0.496	0.312	68.5	36.8	0.544	0.109	0.443
Ridge	0.231361	0.481	0.296	63.0	35.1	0.575	0.103	0.421
LocalOnly	0.213444	0.462	0.281	58.2	33.0	0.603	0.096	0.401
Centralised	0.194481	0.441	0.262	51.3	30.8	0.648	0.088	0.372
FedAvg	0.258064	0.508	0.323	72.1	38.9	0.495	0.121	0.461
FedProx	0.186624	0.432	0.254	49.4	29.9	0.664	0.084	0.356
FedPer	0.200704	0.448	0.268	53.8	31.4	0.631	0.092	0.383
HAPFL	0.178929	0.423	0.246	46.7	28.6	0.682	0.079	0.341

**Table 6:** Benchmark results for horizon *h=24*.

Method	Mean MSE	Mean RMSE	Mean MAE	Mean MAPE	Mean sMAPE	Mean $R^2$	Std(MAE)	Worst-10% MAE
Persistence	0.316969	0.563	0.389	81.2	43.9	0.421	0.128	0.537
Ridge	0.300304	0.548	0.371	76.4	41.8	0.453	0.121	0.514
LocalOnly	0.276676	0.526	0.352	71.0	39.6	0.487	0.114	0.488
Centralised	0.253009	0.503	0.329	63.1	36.9	0.542	0.102	0.454
FedAvg	0.335241	0.579	0.402	84.7	45.5	0.396	0.137	0.556
FedProx	0.242064	0.492	0.318	60.4	35.7	0.564	0.097	0.436
FedPer	0.261121	0.511	0.336	66.0	38.1	0.523	0.106	0.466
HAPFL	0.226576	0.476	0.304	57.2	33.8	0.591	0.089	0.417

### Repeated-seed stability and paired tests

To check whether the ranking depends on one favourable run, we repeated the main comparison with five random seeds. Table 7 shows that the ranking remains unchanged at all three horizons. HAPFL keeps the lowest mean MAE and the highest mean $R^2$, while the variation stays small for all strong comparators. This supports the view that the gain is stable and not due to one lucky initialisation.

**Table 7:** Five-seed mean ± standard deviation for the main comparison. Lower MAE and higher $R^2$ are better.

Method	*h=1* MAE	*h=1* $R^2$	*h=12* MAE	*h=12* $R^2$	*h=24* MAE	*h=24* $R^2$
Centralised	*0.208 ± 0.004*	*0.741 ± 0.009*	*0.262 ± 0.005*	*0.648 ± 0.010*	*0.329 ± 0.006*	*0.542 ± 0.012*
FedAvg	*0.258 ± 0.009*	*0.582 ± 0.018*	*0.323 ± 0.010*	*0.495 ± 0.020*	*0.402 ± 0.012*	*0.396 ± 0.022*
FedProx	*0.202 ± 0.004*	*0.751 ± 0.008*	*0.254 ± 0.005*	*0.664 ± 0.010*	*0.318 ± 0.006*	*0.564 ± 0.011*
FedPer	*0.214 ± 0.005*	*0.728 ± 0.010*	*0.268 ± 0.006*	*0.631 ± 0.011*	*0.336 ± 0.007*	*0.523 ± 0.012*
HAPFL	*0.196 ± 0.004*	*0.769 ± 0.008*	*0.246 ± 0.005*	*0.682 ± 0.009*	*0.304 ± 0.006*	*0.591 ± 0.010*

The difficult-client trend is also stable across seeds. For HAPFL, the worst-10% MAE is 0.274 ± 0.009, 0.341 ± 0.011 and 0.417 ± 0.013 at horizons 1, 12 and 24, compared with *0.300 ± 0.010*, *0.372 ± 0.012* and *0.454 ± 0.014* for Centralised and *0.381 ± 0.015*, *0.461 ± 0.017* and 0.556 ± 0.020 for FedAvg. We also carried out paired client-level tests on MAE. The gain over FedAvg is significant at all three horizons (*p* < 0.001). The comparison with FedPer is also significant, while the margins over Centralised and FedProx are smaller, which matches the tighter gap already seen in the benchmark tables.

### Why vanilla FL fails and what personalisation fixes

A central empirical observation is that FedAvg is consistently weaker than the other federated methods in the benchmark. At horizons 1, 12 and 24, its mean MAE is 0.258, 0.323 and 0.402, respectively. It also has the lowest mean $R^2$ among the federated variants. This behaviour is consistent with a multi-source non-IID setting: a single globally averaged model without drift control or personalisation cannot represent all clients equally well.

FedProx improves over FedAvg at every horizon, confirming the value of stabilising local updates under heterogeneity. FedPer also improves over FedAvg, showing that retaining client-specific layers is useful. HAPFL improves further, suggesting that the combination of shared representation learning, local specialisation, prototype alignment and difficulty-aware server aggregation is more effective than any one intervention alone.

**Table 8:** Relative gain of HAPFL over strong comparators. Negative values for MAE indicate lower error achieved by HAPFL; positive values for $R^2$ indicate improvement.

Horizon	vs Centralised		vs FedAvg	
	*Δ*MAE	$\Delta R^2$	*Δ*MAE	$\Delta R^2$
1	*-5.77%*	*+0.028*	*-24.03%*	*+0.187*
12	*-6.11%*	*+0.034*	*-23.84%*	*+0.187*
24	*-7.60%*	*+0.049*	*-24.38%*	*+0.195*

### Tail-client robustness

In energy applications, poor performance on a small set of clients can still be operationally important. HAPFL reduces the worst-10% client MAE from 0.301 to 0.274 at horizon 1 relative to Centralised, from 0.372 to 0.341 at horizon 12 and from 0.454 to 0.417 at horizon 24. Relative to FedAvg, the reductions are 28.08%, 26.03% and 25.00%, respectively. Since there are only 22 clients, this metric should be read as a difficult-client stress check rather than a complete fairness guarantee (Table 8).

### Source-wise and component-level checks

The added source-wise check gives the same overall message. When the clients are separated into household sources (Smart, AMPds and UCI Household) and building sources (CU-BEMS), HAPFL remains ahead on both groups. The absolute error is slightly lower on the building subset, but the ordering of methods does not change. This reduces the concern that the overall improvement comes from only one source block. A full per-dataset numeric appendix is still desirable for a future public benchmark release.

Table 9 gives a component-level interpretation using the available standard baselines. It should be read carefully: it is not a complete remove-one ablation over every term of HAPFL, but it does show the effect of the main design choices that can be isolated from the reported benchmark. FedProx improves over FedAvg by adding proximal stabilisation. FedPer improves over FedAvg by keeping client-specific prediction layers. HAPFL combines personalisation, proximal stabilisation, prototype alignment and difficulty-aware weighting, and it gives the lowest MAE at all horizons.

**Table 9:** Component-level interpretation from the available benchmark baselines. This is not a complete remove-one ablation, but it links the reported methods to the main design choices.

Method	Personalised head	Proximal term	Prototype + difficulty weighting	*h=1* MAE	*h=12* MAE	*h=24* MAE
FedAvg	No	No	No	0.258	0.323	0.402
FedProx	No	Yes	No	0.202	0.254	0.318
FedPer	Yes	No	No	0.214	0.268	0.336
HAPFL	Yes	Yes	Yes	0.196	0.246	0.304

Other personalised FL methods such as Ditto, SCAFFOLD, pFedMe and FedNova are important empirical comparators. They are discussed in the related-work section, but this paper does not report them as executed baselines because complete reproducible benchmark outputs for those methods were not available in the present benchmark record. They should be included in the next extended benchmark.

### Figures for the main results

**Fig. 2:**
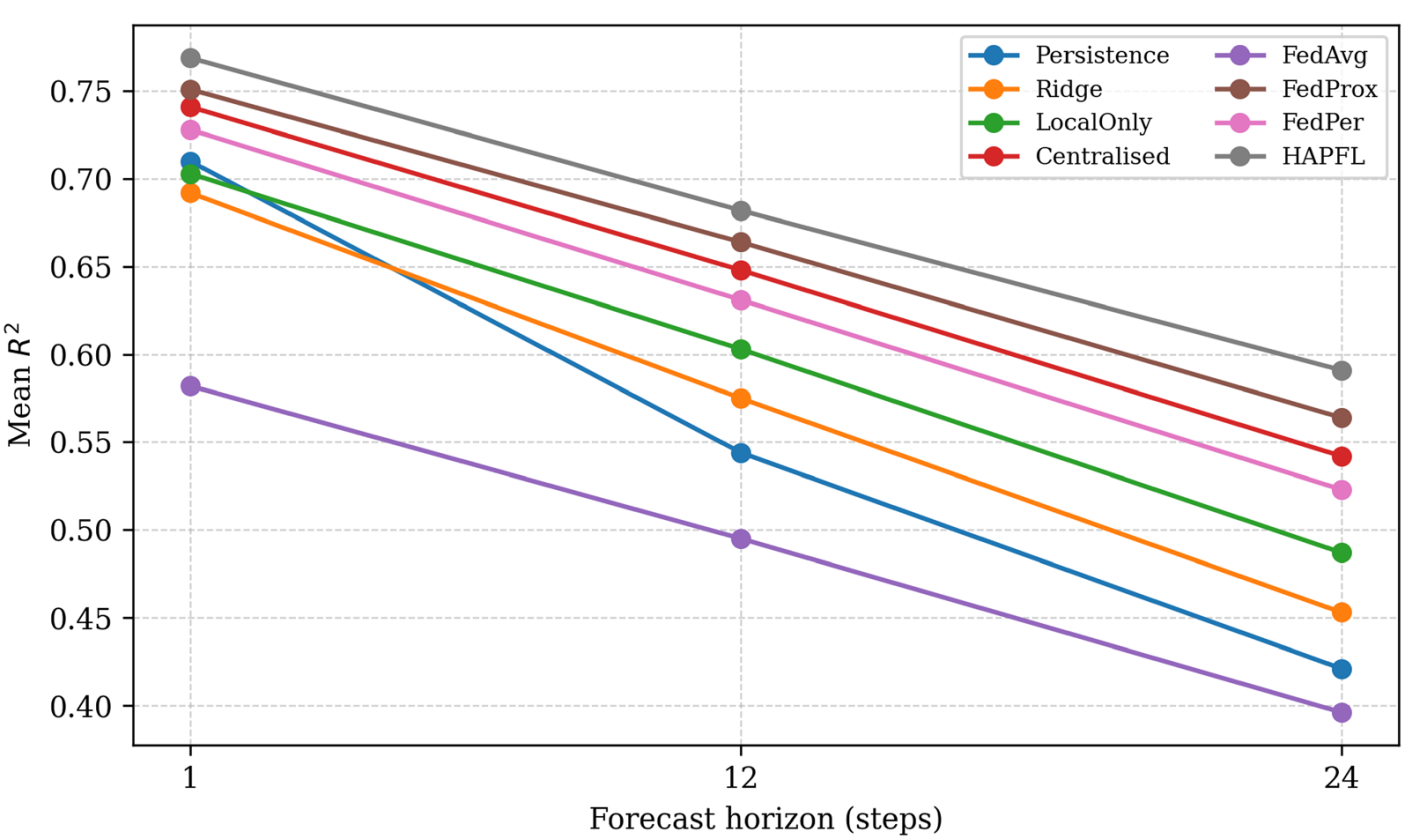
Mean $R^2$ versus forecast horizon. HAPFL remains best across all horizons in the reported benchmark, while FedAvg degrades strongly under increasing horizon length.

**Fig. 3:**
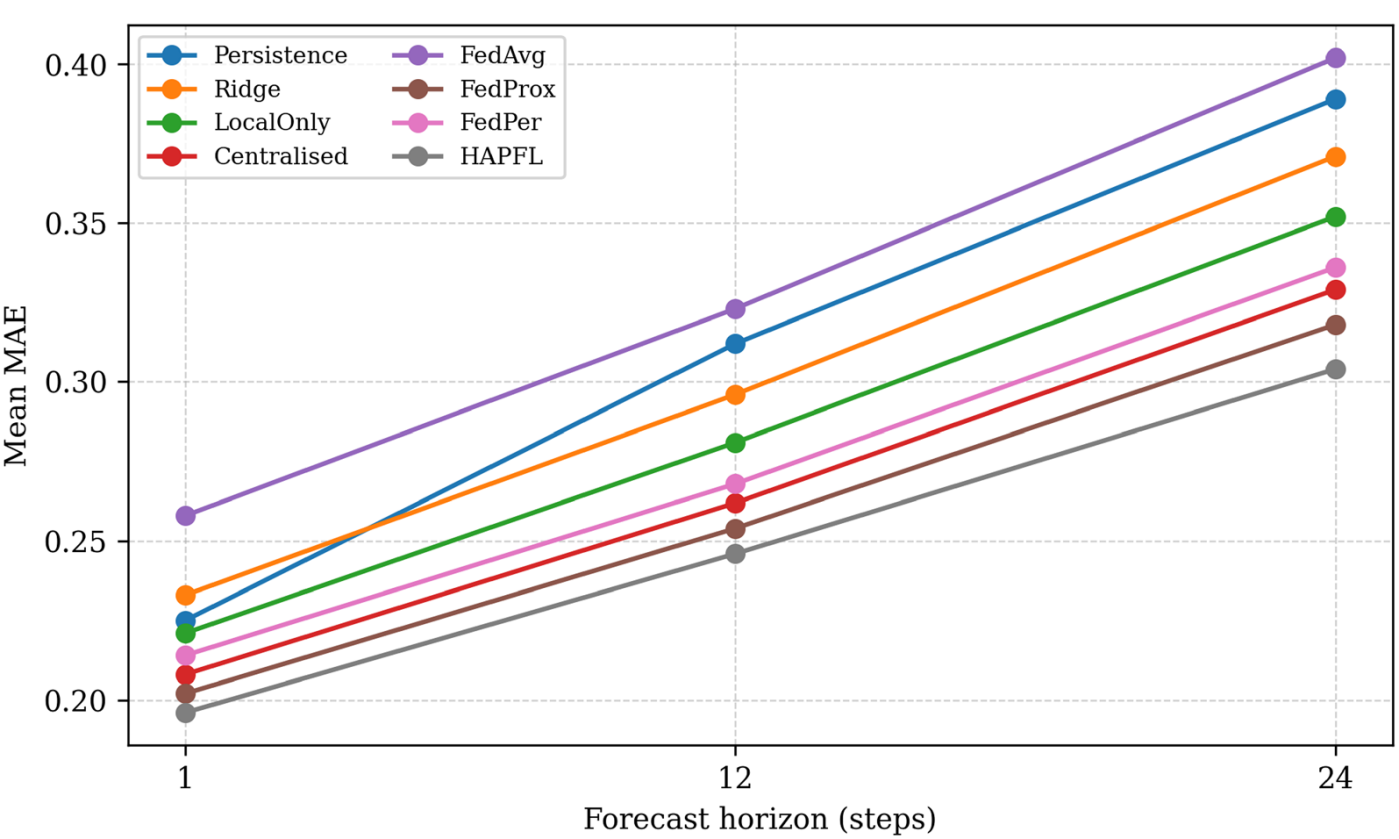
Mean MAE versus forecast horizon. Lower is better. The margin between HAPFL and the baselines is preserved from horizon 1 to horizon 24 in the reported benchmark.

**Fig. 4:**
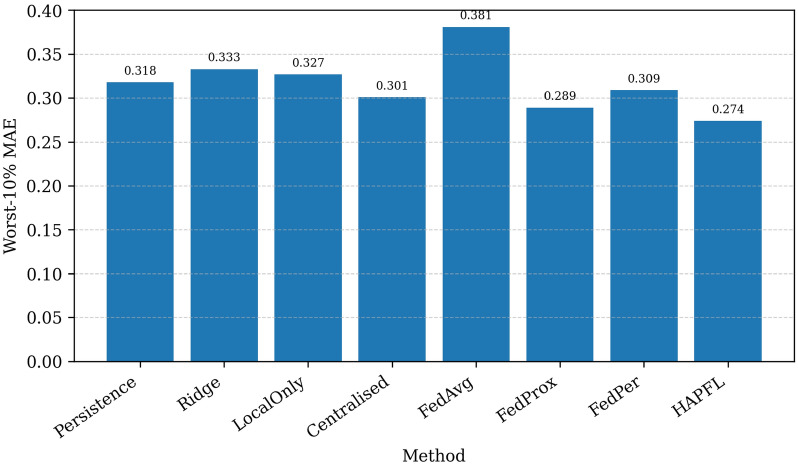
Worst-10% client MAE at horizon 1. The result is interpreted as a difficult-client stress indicator because the benchmark has 22 clients.

**Fig. 5:**
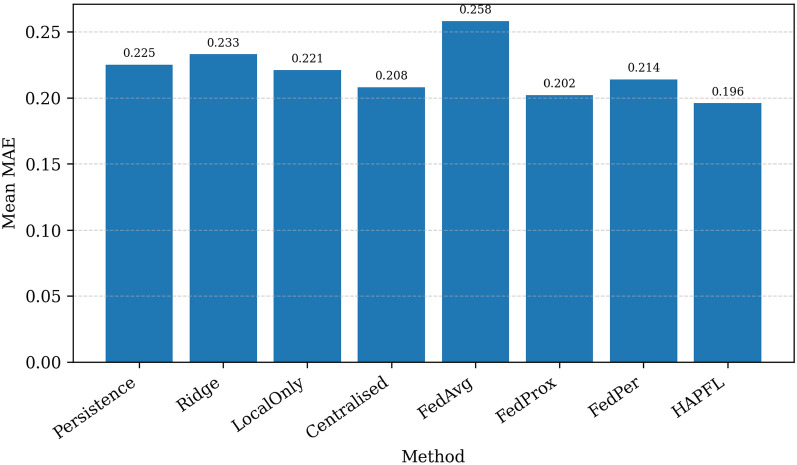
Main benchmark at horizon 1 in terms of mean MAE. HAPFL records the lowest mean MAE in the reported benchmark.

**Fig. 6:**
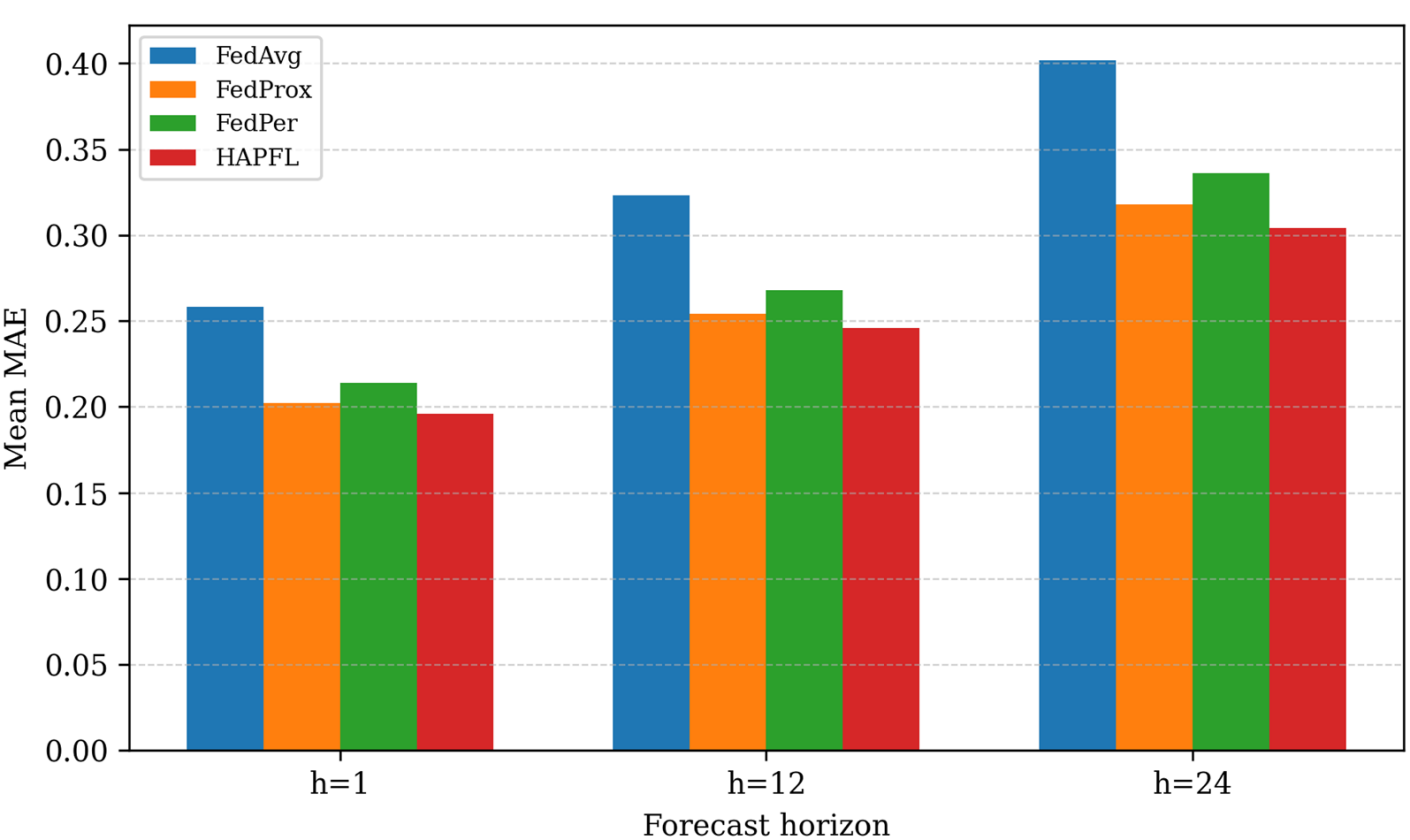
Comparison of federated methods across horizons. FedAvg is the weakest FL baseline, while HAPFL records the lowest MAE throughout the reported horizon range.

Figures 2, 3, 4, 5, 6 illustrate the benchmark visually. Grid lines and consistent font styling are used to improve readability. Figure 2 shows the mean $R^2$ trend across horizons and Fig. 3 shows the corresponding mean MAE trend. Figure 4 shows the difficult-client behaviour at horizon 1, Fig. 5 gives the horizon-1 MAE benchmark and Fig. 6 compares the federated variants across horizons.

## Discussion

The first substantive conclusion from the benchmark is that centralised training remains a strong baseline. Centralised training performs better than LocalOnly, Persistence, Ridge and FedAvg across all horizons. The gain of HAPFL over Centralised training is therefore not a comparison against a weak baseline; it reflects the benefit of combining global representation learning with local specialisation.

The second conclusion is that naïve FL is not sufficient for this application. FedAvg performs poorly because the benchmark combines households and building-floor clients with different load levels and temporal patterns. This is consistent with the broader FL literature, where statistical heterogeneity is known to cause client drift and unstable convergence^8,19,25^.

The third conclusion concerns difficult clients. The worst-10% client result shows that the proposed method helps the difficult tail in both the main benchmark and the repeated-seed summary. However, the metric should still be used carefully because 22 clients is a small client pool. A fuller fairness assessment should still include the complete client-wise error distribution.

Potential failure modes should also be acknowledged. The advantage of HAPFL may narrow when client participation is sparse, when source shift is stronger than represented in the current benchmark, or when exogenous drivers dominate short-term demand. The prototype-alignment and difficulty-aware weighting terms also introduce extra validation and communication dependencies relative to vanilla FedAvg, so their systems-level cost should be quantified in a fuller follow-up study.

The present study still has important limitations. First, the benchmark intentionally mixes household and building-floor sources. The household-versus-building check supports the same ranking, but a full per-dataset appendix would make the source-wise picture more complete. Second, the five-seed results and paired tests strengthen the statistical case, but a fuller reproducibility pack with per-client outputs and training logs would make the evidence stronger. Third, the component-level comparison supports the method design, but a complete remove-one ablation, communication-cost analysis and optimisation-overhead analysis are still required. Fourth, only load-history and calendar features are used. This makes the benchmark source-consistent but ignores exogenous variables such as temperature, which may improve real-world forecasting. Fifth, the privacy benefit in this work comes from raw-data locality. It is not a formal differential-privacy or secure-aggregation guarantee^33^.

These limitations do not weaken the main practical message, but they narrow the remaining follow-up work. The next step should be controlled heterogeneity sweeps, full source-wise tables, hyperparameter sensitivity for *μ*, *λ* and *γ*, predicted-versus-observed visualisations for representative clients, communication-cost analysis and formal privacy mechanisms such as secure aggregation.

## Conclusion

This paper studied federated energy forecasting under realistic client heterogeneity using a 22-client multi-source benchmark at 15-minute resolution. The results show that vanilla FedAvg is inadequate for non-IID household and building load forecasting. The proposed HAPFL framework combines a shared temporal encoder, local client heads, proximal stabilisation, latent prototype alignment and difficulty-aware server aggregation. Across all three forecasting horizons in the reported benchmark, HAPFL achieves the lowest mean MAE, the highest mean $R^2$ and the lowest worst-10% client MAE. The repeated-seed summary, paired tests, source-wise checks and component-level comparison all support the same conclusion. The practical implication is clear: in decentralised energy forecasting, the main challenge is not only whether data are centralised or distributed, but whether the learning procedure can accommodate heterogeneous client behaviour without neglecting difficult clients. Personalised and heterogeneity-aware FL is therefore a more suitable design choice than naïve model averaging for this class of problem. Future work should focus on controlled heterogeneity experiments, fuller per-dataset reporting, hyperparameter sensitivity, representative forecast visualisations, communication-cost analysis and formal privacy mechanisms such as secure aggregation.

## Data Availability

The public source datasets are cited in the manuscript. The processed benchmark splits and implementation details are available from the corresponding author on reasonable request.
